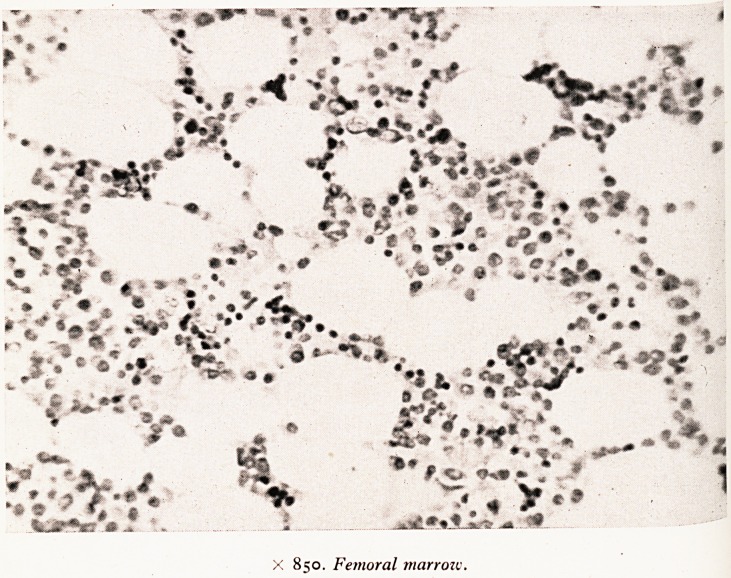# Chronic Osteomyelitis with Maturation Arrest of Granulocytes

**Published:** 1961-01

**Authors:** T. F. Hewer


### CHRONIC OSTEOMYELITIS WITH MATURATION ARREST OF GRANULOCYTES

(*A Clinico-Pathological Conference of the University of Bristol Medical School*) *on 25th October*, 1960, *P.M.* 6571.

CHAIRMAN: PROFESSOR T. F. HEWER

*Mr. J. W. E. Snawdon:* This patient was first seen by me on the 18th August 1959, but I think I should mention that he had been a patient at the Dental Hospital for quite a few years. I have his record card here from which I see he first came under their wing in 1953, so we know that for several years he was an apparently normal patient and had routine conservative dentistry. I think in 1953 he came with a rather neglected oral condition and was persuaded to take an interest in his dentition. He seems to have looked after it reasonably well according to the notes, right up to 1956. At the end of 1956 there is the last entry of any treatment other than the happenings in July 1959. He came up in 1959 with some pain, localized in the left maxilla and swelling, and he was seen as an emergency. It was recommended that his left upper molar should be removed and this was done under local anaesthesia. Nothing was heard of him then, that is from the 7th July when the tooth was removed until the 14th August when he came back again complaining of an unpleasant smell and bad taste in his mouth. He was not feeling particularly well and the resident who saw him at that time noted that a large piece of maxilla was sequestrating and he had possible sinusitis. He was seen again on the 18th August, roughly 5 weeks from the time of the extraction of the tooth in question and on that occasion the patient was referred to me.

The thing that struck me about this man when I saw him was that he was an obviously ill patient. He was very pale and his temperature at that time was not raised at all, but he obviously looked ill and should not be going about in the normal way, working, as he was at that time. When I examined his mouth I saw some bare bone in the left maxilla, but no purulent discharge of note, it was in fact relatively dry, apart from a certain amount of serious exudate on the margins. He had an oro-antral fistula at that stage and a sinus x-ray which was taken showed an opacity of the antral cavity on that side. The main point was that this was obviously an ill patient and the thing we promptly did was to get him admitted to Hospital, and having got him admitted I asked if Professor Perry would be so kind as to see him. At the time however Professor Perry was away and Dr. Leather very kindly came in and had a look at him and carried out the necessary general investigations in the ward. So far as the jaw was concerned we felt there was very little to be done. In general a sequestrum will sort itself out and either present a picture which requires surgical intervention or separate itself spontaneously as often happens. The oro-antral fistula would not present any unsurmountable difficulty subsequently. Now the main point about this man was that he was an obviously ill patient and we felt that the jaw was really a secondary consideration at the time I first saw him. He was investigated and the abnormal blood picture, about which Dr. Leather will have something to say, was discovered. All along we felt that here was the problem which was basically the chief one, an ill patient with this abnormal blood picture where we felt that any surgical treatment of the jaw was contra-indicated and it would not be in his interest to do anything to attempt to remove the dead bone. We felt that the jaw problem then was undoubtedly secondary to his systemic condition. In other words that he had this condition present when the tooth was extracted and the tissues reacted in this way because of the nature of his blood condition at that time. There is one other point I would just like to clear up, and that is to say he improved a lot on his general treatment. This went on from August 1959 until December 1959, when he was not quite as good but on the whole was much the same as in the early stages; I was always expecting that as his general condition improved the piece of bone would sequestrate, but on the 19th January 1960 he came in and said he had come prior to the next routine check that had been arranged because he was getting some discomfort in his upper jaw and also some more offensive discharge. He was obviously looking very bad, he had quite a lot of boils around his face, he looked very pale again and obviously ill, so I wrote to Professor Perry as follows "This mutual patient of ours came to see me to-day. I do not feel very happy about his condition, his pallor has increased again since I saw him six weeks ago and he is being troubled by an offensive nasal discharge. The amount of necrotic bone visible in the mouth has increased and the oro-antral fistula is of course still present. Whilst I feel that any surgical intervention is at present contra-indicated in this case I wonder how you now regard his general medical condition also the possibility of removing the necrotic bone some time in the future? I think if left to its own devices it will separate but it is likely to prove rather troublesome to him." And Professor Perry in his reply said "We saw this patient at the end of December, his haemoglobin was then just under 70 per cent. If he is getting worse perhaps we should have him into hospital again and we would ask your advice when he is in".

That time when I saw him in January was, in fact, the last time that I actually examined him and the next thing I knew was the post mortem findings.

*Dr. H. M. Leather:* You have heard quite a lot about this patient already and I shall not emphasize any of the dental aspects of his illness. He was a man 60 years of age, and was a goods porter. In the previous history he had had a prolapsed intervertebral disc in 1952 for which a laminectomy was performed, and after a certain amount of subsequent pain at that period the trouble cleared up and did not return. He had had an attack of bronchitis two years before his present admission.

In March of last year, three months before the tooth socket trouble he attended the Casualty department with a septic finger. He was given some glycerine and mag. sulph. paste and also given an injection of 1 mega of crystalline penicillin. Following this he fainted. He was accordingly detained in Casualty for an hour or so before being allowed to leave. There were no sequelae to this curious episode, he developed no skin rashes, and in retrospect we were inclined to think that this was a faint that might have followed any injection. We first saw him in August 1959, Mr. Snawdon having referred him. He told us that he had lost several pounds in weight over recent months but that was the only addition to the recent history. He looked ill, the mucous membranes were pale, the spleen was impalpable, the lymph glands did not appear to be enlarged, and there were no petechiae. Hess's test was positive. A blood count was done and revealed haemoglobin 58 per cent, the red blood cells showed considerable anisocytosis with some macrocytes present. There was generalized hypochromia. Total white cell count was 3,200/c.mm. and only 680 of these were polymorphs. The platelet count was 120,000/c.mm and the sedimentation rate was 44 mm. in the first hour. These findings, with a decrease in the red blood cells, white blood cells and platelets, suggested we were dealing with a marrow hypoplasia and accordingly a sternal marrow was carried out by Dr. Raper. This showed that the degree of cellularity in the specimen examined was normal and that only slight changes in the myeloid series were present. Dr. Raper felt that this marrow picture might have represented recovery from a hypoplastic phase. Now hypoplasia and aplasia of the bone marrow may result from toxicity to a variety of substances. Among the many drugs which can do this are the heavy metals such as gold and mercury, the antibiotic chloramphenical, the sulphonamides, thiouracil and its derivatives, tridione and various substances used in industry. The number of drugs which can do this is increasing all the time. The patient was questioned very closely to determine whether in fact any such drug had been given but there was no evidence that he had taken anything that might have caused hypoplasia of his marrow.

We took a culture from the tooth socket to see if there were any pathogenic organisms present; we did not think it very likely that there would be, and in fact we grew a mixed flora with alpha haemolytic streptococci predominating, as one might expect from the mouth. We gave him a course of tetracycline, on the assumption that by giving him a wide-spectrum antibiotic we might improve the condition of his tooth socket, but it had little effect except that his temperature, which had been a little raised to 99° on admission, settled during that course.

We also gave him a blood transfusion, 3 pints of blood in all, which improved his haemoglobin level. When the blood was grouped it was found that it was rather difficult to determine which group it was. It appeared that it might have been group O or A. In fact it was concluded that it was probably group A but this difficulty with the grouping raised the possibility that we might here be dealing with aleukaemic leukaemia rather than an ordinary marrow hypoplasia. He went home after a month and his condition was pretty well unchanged. The haemoglobin had risen to 83 per cent following the blood transfusion but the tooth socket remained more or less the same. The white count had remained very low, the lowest level recorded being a total of 1240 white cells/c.mm. with only 140 polymorphs. He said he felt better however and he certainly had gained a little weight.

He was seen a couple of weeks later in Outpatients and at that time his condition was more or less the same, but he said that on two occasions some brown fluid had come down his left nostril. It was thin watery fluid, and there had been a few cc. on each occasion. We attributed this to antral involvement which would not be surprising as the tooth socket affected was in the left upper jaw. In fact as Mr. Snawdon has told you there was an oro-antral fistula present. There was no clinical evidence of any acute process going on, there was no tenderness over the antrum and we did not do anything about this discharge.

About 7 weeks after that, in November of last year, he was re-admitted with a boil on his cheek, and also quite a severe blepharitis. The physical signs at this time were more or less the same, the tooth socket being pretty well unchanged. We took a swab of the conjunctiva and grew a *Staph. aureus* which was fully sensitive. The blood count was substantially unaltered and another bone marrow biopsy showed very little change from the first. On this occasion also we gave him a course of tetracycline and we tried to prevent further staphylococcal infection by giving him neomycin cream to put up his nose and hexachlorophene dusting powder for the perineum, both sites well known to be areas of staphylococcal proliferation. He also had hexachlorophene in the bath water for the same purpose. The lesions resolved and he went home.

On the 19th January he was seen by Mr. Snawdon and was subsequently admitted under the care of Dr. Barritt at Ham Green Hospital in March. His general condition was again pretty well unchanged. He complained of some pain over the left side of the back of the head and there were some glands in the posterior triangles which were thought to be enlarged, though not painful. Well, once more the bone marrow was examined. There were again relatively slight differences about which we shall hear, but they did raise the possibility that this might be myelomatosis and so his urine was examined for Bence-Jones protein and his serum proteins were electrophoresed to see if there was any of the abnormal myeloma protein present. In fact there was no Bence-Jones in the urine and though the plasma proteins did show a slight rise in the gamma globulin there was no abnormality other than that. He was again given a blood transfusion of 2 pints of packed cells, and a course of tetracycline followed by a course of erythromycin. He was also (I think on Mr. Snawdon's recommendation) given hibitane lozenges to suck. The blood transfusion raised the haemoglobin level from 60 per cent on admission to 77 per cent when he went home.

On the 11th May, about 9 months after we first saw him, he was admitted for the last time. He had been found semiconscious at home by his doctor who had gone to see him. On admission he was barely conscious and could obey simple commands but was unable to give any history. There was purpura with ecchymoses in the skin. He had no obvious photophobia but there was marked neck stiffness. The pupils were small, reacting only sluggishly to light. His haemoglobin was 56 per cent and on this occasion the white cell count was 8,000/c.mm. with 6,200 polymorphs. A lumbar puncture was done and the pressure was 180 mm. of water. Queckenstedt's test was normal. The cerebro-spinal fluid was turbid, of a greenish-grey colour, and contained 7300 white blood cells/c.mm., more than 90 per cent of which were polymorphs. The protein was raised, 870 mgm per cent, and the sugar had fallen to 13 mgm per cent. A stained film of c.s.f. revealed no organisms and the culture was sterile. He was treated with chloramphenicol and streptomycin but it made very little difference. The temperature which had been normal on admission rose steadily to 102°F. during the 2 or 3 days before he died. His general condition remained unchanged for about 24 hours but then went downhill. On the 14th May, 72 hours after admission he vomited, I think some vomit was aspirated and he died shortly afterwards.

To summarize, this was a man of 60 in whom a tooth socket failed to heal after dental extraction. He was found to have a hypoplastic anaemia, the cause of which was uncertain. He showed increased susceptibility to infection and died 11 months after the tooth extraction as a result of an infection, in fact of meningeal involvement. Throughout his illness the anaemia and leukopenia had persisted.

*Dr. A. B. Raper:* For the ten months or so during which this man was under observation he remained an unexplained haematological problem and in fact he fell into the group which contributes most to unexplained haematological problems, that of pancytopenia, a reduction of all the formed elements of blood. Dr. Leather has spoken of the findings when he first came into hospital. At that time he had only 680 polymorphs/c.mm. and his haemoglobin was 53 per cent. But only two days before, when he had been sent across from the Dental Hospital by Mr. Snawdon, his polymorphs had been 2700 and haemoglobin was 63 per cent, so it looked at that time as though quite rapid changes had been occurring, and he had been suffering a rapid fall in his red and white cells. When the bone marrow was then examined it was found that myelocytes were rather more numerous than segmented cells, and that was the finding that Dr. Leather mentioned. We thought this was a change of relatively recent origin and that he was perhaps beginning to recover from a temporary depression of granulopoiesis, and that only the future would tell whether this was so or not; but the future revealed that it was not so, for he remained pancytopenic throughout, apart from the influence of transfusion. Marrows were examined at long intervals, the first in August, the next in November and the third in March. That in November showed a similar picture to the first, but the myelocytes were rather more numerous, the segmented polymorphs were still scanty, and there were more promyelocytes. There was obviously a left shift occurring in the marrow to rather more primitive forms, and there were some unidentified cells on that occasion. In March the whole picture had shifted a little more to the left. There was now a sizable number (8 per cent) of promyelocytes, the myelocytes were very numerous (25 per cent), and were accompanied by 8.5 per cent of other cells which somewhat resembled them and which gave rise to some difficulty in classification. It was these cells that made us think of the possibility of plasmacytosis, for in some ways they resembled plasma cells. As you have heard that possibility was negatived by the finding that there was no Bence-Jones proteinuria, X-ray of the bones showed nothing, and the serum protein pattern was normal. I eventually concluded that these abnormal cells were micromyelocytes, and this label does raise the question of whether they were actually abnormal leukaemic cells, because one does sometimes see abnormal small myelocytes in the blood and also in the marrow in myeloid leukaemia. In summary, a maturation defect had been observed throughout, a piling up of the myelocytes with failure to develop any further, and this became more noticeable as the months passed. Nevertheless at the end of these 3 marrow examinations I wrote: "there is no direct evidence of leukaemia". Whether there was evidence of a change in the direction of leukaemia, and what the bearing of these three marrow investigations on this evidence is, I don't know. I think we had better delay a firm decision until Dr. Tovey has given his views.

*Dr. G. Tovey:* A sample of this patient's blood was sent to the Regional Transfusion Centre because of the difficulty of deciding whether his blood group was A or O. Now briefly, if you want to decide whether someone is group A you add the patient's red cells to an anti-A grouping serum. If the cells are clumped the patient is group A: if they are not agglutinated the patient is group O. This man's red cells were clumped in such a test so that it looked as if his group was A, but at the same time a large number of the red cells present were just not clumped at all. Those might well be group O cells; hence the difficulty in deciding whether his group was O or A.

Now what might be the cause of this unusual phenomenon? Clearly one might be dealing with a group A patient recently transfused with group O blood. Fortunately, however, the specimen coming from this patient was taken before he was transfused. One might therefore be dealing with a blood group chimera. I am sure you know the chimera, a mythical animal with the body of a goat, the tail of a serpent and the head of a lion. A blood group chimera is a twin who has received some of his fellow twin's blood-forming tissue whilst *in utero*. Two types of haemopoetic tissue are therefore present in his body. One may be forming group A cells and the other group O cells, and so this man might have been a chimera. We were able to exclude this, not only because he denied being a twin, but also by testing whether we could separate the A cells from the O cells. In the case of a chimera, if you add the cells to anti-A all the group A cells will be precipitated and the group O cells left suspended. In the case of this patient however we could not separate the apparent mixture in that way. Thirdly, his blood group might be one of the unusually weak sub-groups of A, the so-called weak A's, ($A_3$, $A_4$, $A_5$, etc.). Blood group substances are not confined to the red cells but are present also in the saliva. An individual who has a weak A in his red cells will similarly have only a small amount of group A substance in his saliva. The patient's saliva was found to contain as much group A as any normal group A individual. The last possibility remained therefore that one was dealing with a patient whose blood group had been changed as a result of disease. This type of change has been seen only in acute leukaemia and in our report to Professor Perry therefore we queried whether this patient might be suffering from an aleukaemic leukaemia.

*Dr. W. A. Gillespie:* This cerebro-spinal fluid was seen by the resident pathologist during the night, and I saw it the following morning. It was a good sample, with plenty of fluid, and was obviously turbid with numerous polymorphs, as you have heard. The glucose content was quite low, 13 mgm per cent, and this rather suggested that the condition was a diffuse meningitis and not simply a sudden rupture of a brain abscess, which can also give a purulent fluid. However, it proved impossible to find any organisms in the stained smears of the deposit although we examined several slides. We knew that the patient probably had a lowered resistance to infection, as a result of his blood disease; and so we thought that the meningitis might have been caused by an unusual organism which normally would be unlikely to cause meningitis. We had in mind, for example, *Listeria*, which may look like a diphtheroid, and sometimes causes infection in cases like this. We also thought of fusiform bacilli and other members of the *Bacteroides* group, in addition to the organisms which more usually cause meningitis. We therefore put up several cultures anaerobically as well as aerobically and incubated them for several days at 37°C. But all the cultures were sterile. At first we thought that the absence of demonstrable organisms might be due to recent antibiotic treatment, before the patient entered hospital; but this was not the explanation. He had not received any drugs before admission. At the time I did not think that the failure to find an organism mattered very much because the patient was treated energetically with a mixture of antibiotics which should have been active against any bacteria.

*Dr. O. C. Lloyd:* This man was wasted. In the serous cavities there was no excess of fluid but there were some old fibrous adhesions at the upper part of the right lung, and in the right lung I found an old healed primary tuberculous complex, with signs of healed tuberculous pleurisy. The heart showed well marked fatty change of the inner third of the myocardium which may have been due to his anaemia. The arteries and veins were all right, but I did note that the post-mortem clot in the veins had separated out and had a very small red component and a large white component; that is evidence of anaemia. The spleen was not enlarged, it was slightly soft and the pattern was normal. He did not have one of those acute splenic tumours you sometimes get with inflammation. The lymph nodes were normal with the exception of one high up in the left cervical chain, which was enlarged. That may well have been the one which Mr. Snawdon mentioned and it was probably reactive hyperplasia from the inflammation which had been going on in his maxilla. The liver, gall bladder, and pancreas were all right. The whole of the gastro-intestinal tract was natural and so also were the urinary system, the genital system and his endocrines. So now let me show you what was wrong with him.

A close-up photograph of the tooth socket in left maxilla (Plate I) shows a certain amount of necrotic bone in the middle of the ulcer. At post mortem I dissected out the anterior part of the left hand side of the face and cut a slice through it in the coronal plane of the body so that it went through the ulcer. The maxillary air sinus has a great thick oedematous lining to it. There is a little bit of swelling and discoloration of the medial side of the orbit where the osteomyelitis which was affecting all of these bones to a greater or lesser extent was rather more acute than elsewhere. It was bursting through into the orbit and into the frontal sinus on that side. All the same I have no doubt that the frontal sinus had been inflamed for as long as the maxillary antrum.

The posterior part of the left frontal sinus was seen to be filled with sticky yellowish necrotic debris. The other frontal sinus was similarly affected and the inflammation spread, through its posterior wall, forming a collection of similar yellowish necrotic material in the mid-line on both sides, just outside the dura mater. On the right hand side this inflammatory process had extended back through into the cranial cavity. The infection had travelled along one of the little emissary veins which goes between the meningeal vessels at that point and the cerebral veins.

A section shows that the cortex of the maxilla is eroded at the point at which the tooth has been pulled out and there is a lot of new bone formation. In the necrotic surface of the tooth socket I found an organism with the morphological appearance of a ray fungus or actinomyces. It is quite likely a pathogenic species of *Actinomyces*.

Plate II shows in the tooth socket a piece of dead bone which would have been sequestrum, if he had been able to get rid of his dead tissue, but he was not able to do so. He had very good healing powers. The squamous epithelium has been growing back all round the fragments of dead bone; the submucosa contains lymphocytes and small round cells, but hardly any polymorphs. "You must get rid of the dirty water before putting in the clean". If you have an inflammation you must get rid of the necrotic material before any healing process can be adequate, and in order to get rid of necrotic material you have to have both active reticulo-endothelial and haemopoietic systems; and *that* was what this man lacked. You must have polymorphs, phagocytes and all other elements working properly or you cannot get rid of necrotic tissue of this sort and the healing process will not be effective. There has been some attempt to remove the bone, since there is a little bit of lacunar resorption, but it has not been very effective.

Deep within the maxilla there is chronic osteomyelitis, while in the wall of the maxillary antrum there is a thick layer of oedematous submucosa which contains a

PLATE I

*Tooth socket in left maxilla.*

PLATE II

× 100. *Surface of tooth socket.*

PLATE III

*Brain: transverse slice. Abscess in right frontal pole.*

PLATE IV

× 850. *Femoral marrow.*

fair number of macrophages and other small round cells and just an occasional polymorph here and there. There has been a lot of new bone formation in the lining of the antrum.

In the wall of the left orbit the osteomyelitis is rather more acute, and the connective tissue is very much more vascular. It is granulation tissue and contains lymphocytes and very many more polymorphs than elsewhere. Although this man did not have enough polymorphs to go round he did have some, and they tended to congregate in those places where they were most needed although there were probably not enough of them. Where the inflammation is more acute he was able to do something about destroying his deficient tissues. There is a considerable amount of lacunar resorption going on round these bone fragments.

The frontal sinus epithelium has undergone squamous metaplasia which is a reaction to chronic inflammation. There is considerable formation of new bone under the submucosa. The inflammation crept along one of the communicating veins and entered the brain. Looking at the brain from the outside there was flattening of the convolutions and when it was turned upside down, pus could be seen in the interpeduncular region and around the stalk of the pituitary body. That pus was a greenish colour but the green faded after fixation. It was not the kind of green you get in a *pyocyanea* infection, and was probably due to the presence of inflammatory leucocytes in the pus.

Plate III shows a transverse slice through the brain. A glass rod has been inserted through the hole in the right frontal pole. It enters the cavity of the abscess. In the necrotic wall there are large numbers of petechial haemorrhages. The abscess has burst into the ventricular system giving rise to a generalized ventriculitis. The inflammation went right through the fourth ventricle and out through the basal foramina and so into the meninges below. It was from a septic meningitis that he was suffering and that was in fact the cause of his death. The wall of the abscess microscopically showed a frayed free edge with practically no pyogenic membrane. It was just a quiet necrosis. There was a zone of inflammatory cells and a close examination of these cells showed they were more or less half and half polymorphs and macrophages. He was able to produce some reaction but very little.

I looked at a Gram stain of this section and was unable to detect any bacteria but did see something which I considered to be mycelial fragments. I also looked at a Gram stain of the necrotic material in the frontal sinus and there there were distinct mycelial fragments from a fungus but it is quite impossible to tell what kind of fungus it might have been. You cannot tell that unless you can see the fructification and even then you may not be able to be quite certain. It might have been actinomyces or it might have been monilia. The other thing which I saw in the Gram stain from the frontal sinus necrotic material were some rather small and miserable-looking gram positive cocci which were growing in short chains. I do not know whether they had much to do with it, at any rate Dr. Gillespie did not manage to grow it from the c.s.f. during life nor from the post mortem pus from the meninges.

Bisection of the femur showed bone marrow undergoing hyperplasia. A man of this age ought to have yellow marrow practically throughout his femur, but here the upper half of it is red so that it does not look like a marrow aplasia at all. Plate IV shows a considerable degree of reactive hyperplasia in the femoral marrow. Most of the primitive cells are represented but not very many mature elements, in particular there are very few mature pclymorphs. The primitive cells include haemocytoblasts and large numbers of myelocytes. There are a few plasma cells and eosinophilic myelocytes are fairly conspicuous in some places. There are numerous macrophages containing iron pigment. Evidently he had been destroying blood rather more rapidly than usual.

*Professor Hewer:* There was only one granule resembling actinomyces in all your section?

*Dr. Lloyd:* Just one fragment in the tooth. That was the only fructification anywhere.

*Professor Hewer:* That might very well be where it commenced, if it was actinomycosis.

*Dr. Lloyd:* It might, yes.

*Mr. Snawdon:* I would not say there was anything in this case to make one think clinically of actinomycotic infection. Perhaps I might now clarify two points I should have made earlier: I noticed on his dental record he has a red stamp marked "Idiosyncrasy to penicillin" and although it has no date, judging from the writing and the general wear and tear it has suffered I would put it at about the years 1954–1956. And the other final point: I said the 19th January was the last time I saw the patient for clinical examination. That was true but I should have mentioned that Dr. Barritt did telephone me just after he had come under his care, a few weeks after the last time I saw him that was, to ask one or two points and my views about the case, which were unchanged at that time of course.

*Professor Hewer:* I take it then that one of the chief clinical points against actinomycosis was absence of numerous sinuses externally.

*Mr. Snawdon:* There was just nothing. I do not think I have ever seen actinomycosis affecting the mucous membrane. It involves the skin externally when it appears associated with the jaws and face in my experience, and without a demonstrable bony lesion.

*Dr. Gillespie:* I do not think that this was at all like actinomycosis. We occasionally see cases of actinomycosis, mostly from the Dental Hospital, and in the material one finds colonies of the organisms, the so-called "sulphur granules". There were no such granules seen anywhere in the sections from this case, apart from the one in the tooth socket.

The failure to isolate a bacterium at post-mortem was of course not surprising because the patient had been energetically treated with antibiotics. It was, however, surprising not to find an organism during life before the antibiotic treatment started. I take it that the condition on admission to hospital was a recent rupture of an abscess which had slowly formed in response to invasion by organisms of very little pathogenicity which had died out to a large extent.

*Dr. Lloyd:* Would that apply to a fungus infection of the brain?

*Dr. Gillespie:* I don't know. I have never seen a fungus infection of the brain. But do you mean an actinomycotic infection?

*Dr. Lloyd:* Or a monilial, yes.

*Dr. Gillespie:* I do not think this was a monilial infection. A monilial infection should have been detectable in the post-mortem material. And the organism would have been easy to isolate. Another point is that fungi and monilia (but not actinomyces) are insensitive to the antibiotics used in this case, and should not have been inhibited by them.

*Dr. Leather:* Would an actinomycosis, even if it did break out from an abscess, result in an acute picture like this, or would it not be more chronic?

*Dr. Gillespie:* I think that any abscess, irrespective of its cause, would produce an acute picture if it ruptures and spreads over the meninges.

*Dr. Leather:* I was just wondering whether an acute process would result in the case of actinomycosis.

*Dr. Gillespie:* The rupture of an actinomycotic abscess would produce an acute picture, I think.

*Dr. Tovey:* Five cases have been described showing this type of blood group change and in each case the patient has died of acute leukaemia. One of those patients presented as a pancytopenia, thus closely resembling this patient, but eventually died of acute leukaemia.

The May number of the American Journal of Medicine (1960) contained an interesting article by Carl Moore on the subject of pre-leukaemia, and includes an account of a patient who suffered a refractory anaemia for nearly ten years during which he received 50 blood transfusions, and then terminally his haematological state changed suddenly to that of an acute leukaemia. Moore stresses that in pre-leukaemia lesions of the mucous membranes fail to heal satisfactorily. The haematological picture may be that of aplasia of the marrow, or there may be a relatively hyperactive marrow similar to that seen in this case at post mortem. The spleen is not enlarged, neither is there any enlargement of the lymph nodes or liver until the terminal acute leukaemia. I would like therefore to suggest that this man was suffering from a pre-leukaemia and that, had he been able to hold on to his tooth, he might have lived sufficiently long to have presented as a case of acute leukaemia.

I think it is a very important case in that, if it is a fact that his was a pre-leukaemic condition, it is instructive to see that the blood group change may be present long before the typical bone marrow changes of acute leukaemia. If a cure is one day found for acute leukaemia, clearly the earlier one can diagnose such cases the better.

*Dr. Shaw:* May I ask Dr. Raper whether in view of the post mortem findings of hyperactive marrow, he would put this man in that interesting group of refractory anaemias with a hyperplastic marrow? Secondly, could I ask for clarification from Dr. Tovey about why you cannot separate the group A cells from the group O cells?

*Dr. Raper:* It is true that as he went on his red cell series became more active in his marrow, and in that respect his marrow became hyperactive. But I do not think that was the fundamental change in this case.

Since there has been so much discussion about "pre-leukaemia" I think it might be a good thing to clarify this. What do we mean by pre-leukaemia? We may mean several different things. We can mean a state which inevitably leads on to leukaemia. Now no one has ever detected such a state, and we cannot say with certainty that a given person who has now not got leukaemia will later develop it. Secondly, we can mean (at the other end of the scale) a whole group of conditions which may in some people, and with a small degree of probability, terminate in leukaemia; polycythaemia for instance, and some cases of pernicious anaemia fall into that group. Thirdly we may apply the term to those people who, with a rather greater degree of probability, will become leukaemic. This last is the group into which this patient falls, with a maturation arrest in his marrow and a pancytopenia, for we do know that if we cannot explain this state on other grounds then there is a fair probability that it will eventually prove to be leukaemia. Now what is so interesting in Dr. Tovey's presentation is that he may be succeeding in separating some members of this group and actually putting them into the group No. 1, with an almost complete certainty of development of leukaemia, and so we may find that the application of this test may open up group No. 1, and as he says open up also the possibility of early treatment.

*Dr. Tovey:* (in reply to Dr. Shaw), The cells which we have been describing as group O are, in fact, not group O cells. They are group A cells modified by disease. As you know, normal group A cells are clumped together by an anti-A serum. These modified group A cells are not clumped by anti-A, but they do absorb anti-A and, as a result, cannot be separated artificially in the test tube.

*Professor Hewer:* And it is in a case like this where the cells, presumably modified A, are left over, that you are able to show the presence of the A antibody on the surface?

*Dr. Tovey:* Yes, the A antigen was there and the anti-A could be eluted from the red cells after attempting to agglutinate them with an anti-A grouping serum.

*Professor Hewer:* And did you in fact elute off the anti-A?

*Dr. Tovey:* Yes.

*Dr. Leather:* If this very curious change appears with any frequency in aplastic or hypoplastic anaemias would we not in fact know about it, as most of them are grouped for the purpose of transfusion?

*Dr. Tovey:* We are investigating this problem with Dr. Raper's help. It is interesting that after we published a similar case from Bristol two years ago when such a change was considered a rarity, I received several letters from continental blood group workers, and from the States, saying that they are quite certain they have had cases like this in the past but had decided that the patient had a weak sub-type of group A. There is no doubt that these cases have been missed in the past, but again if you have a significant proportion of normal A cells in the mixture and you are not looking very carefully you will mis-group that person as a normal group A.

*Dr. Raper:* Do you think that such changes are inherently more probable if the patient possesses the A antigen? Of course they are more readily detected in such a case, but are there any means of detecting a change if the patient's blood group is originally O?

*Dr. Tovey:* No. I can think of no means at the moment if the patient starts group O. One of the odd features is that in all cases the patient has been group A.

Just as a foot note, we are investigating a patient in North Devon, with chronic leukaemia, whom we think has an essentially similar change to this, but in two of her Rh antigens, D and E. Should this prove to be so, this patient is unique as such a change has not been seen before in the Rh groups. It is interesting that the underlying condition is chronic leukaemia and not acute.

*Professor Hewer:* I have an idea I read recently that leukaemia was commoner among group A than group O. Is that true?

*Dr. Tovey:* Not really established. Another series has been published which did not substantiate that claim.

*Dr. McConnell:* Dr. Tovey, did you determine this patient's Rh group?

*Dr. Tovey:* Oh yes, all the other blood group factors were thoroughly investigated and there was no change in any other blood group antigen.

## Figures and Tables

**Figure f1:**
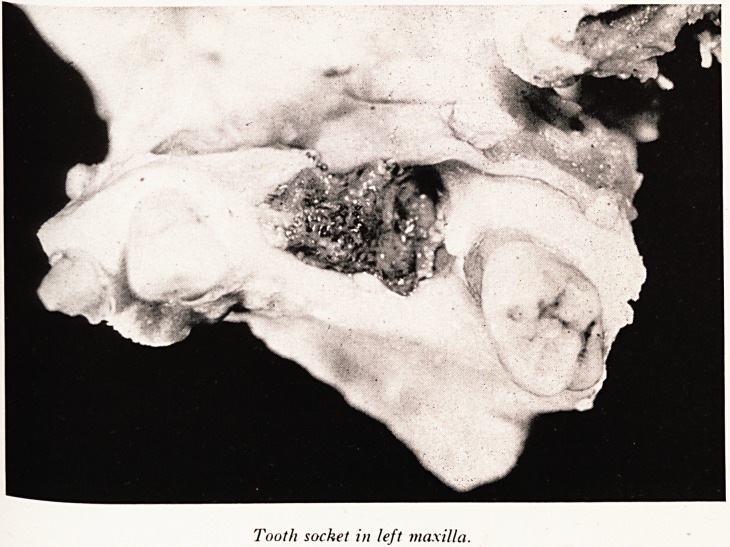


**Figure f2:**
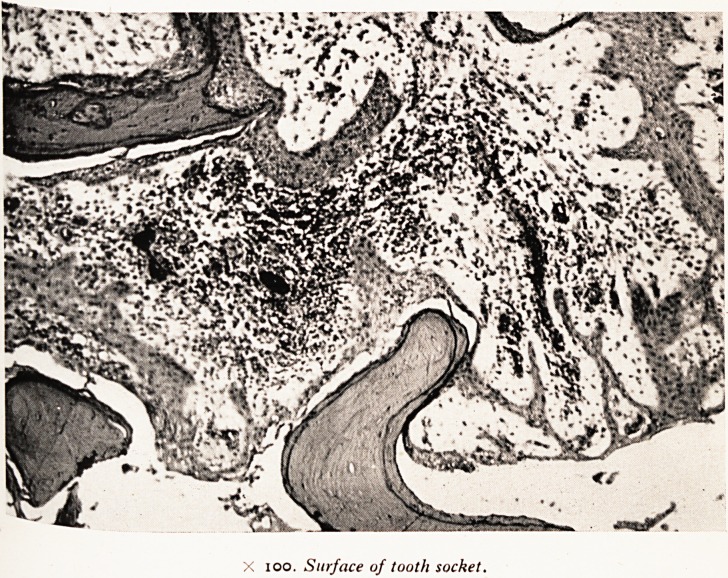


**Figure f3:**
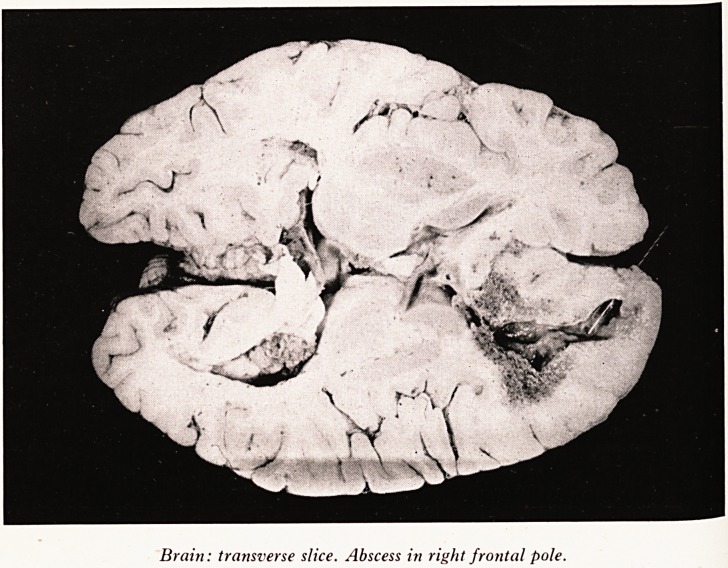


**Figure f4:**